# Polyphenolic Composition and Antimicrobial, Antioxidant, Anti-Inflammatory, and Antihyperglycemic Activity of Different Extracts of *Teucrium montanum* from Ozren Mountain

**DOI:** 10.3390/antibiotics13040358

**Published:** 2024-04-14

**Authors:** Pero Sailović, Božana Odžaković, Darko Bodroža, Jelena Vulić, Jasna Čanadanović-Brunet, Jelena Zvezdanović, Bojana Danilović

**Affiliations:** 1 Faculty of Technology Banja Luka, University of Banja Luka, Bulevar vojvode Stepe Stepanovića 73, 78000 Banja Luka, Republic of Srpska, Bosnia and Herzegovina; pero.sailovic@tf.unibl.org (P.S.); bozana.odzakovic@tf.unibl.org (B.O.); darko.bodroza@tf.unibl.org (D.B.); 2 Faculty of Technology Novi Sad, University of Novi Sad, Bulevar cara Lazara 1, 21102 Novi Sad, Serbia; jvulic@uns.ac.rs (J.V.); jasnab@uns.ac.rs (J.Č.-B.); 3 Faculty of Technology Leskovac, University of Niš, Bulevar oslobodjenja 124, 16000 Leskovac, Serbia; jzvezdanovic@tf.ni.ac.rs

**Keywords:** *Teucrium montanum*, bioactive compounds, antimicrobial potential, DPPH, α-glucosidase inhibition, protein denaturation inhibition, UHPLC-MS

## Abstract

*Teucrium montanum* has widespread use in folk medicine on the Balkan peninsula. In order to scientifically justify this use, the composition and biological activity of aqueous, ethanol, and acetone extract were investigated in this study. Moreover, acetone and ethanol extracts were obtained from the plant material previously exhausted by water extraction. A total of 27 compounds were detected in extracts by UHPLC-DAD-MS/MS analysis, with all of them present in acetone and ethanol extracts. Consequentially, the acetone and ethanol extracts showed higher contents of total phenols of 23% and 18%, respectively, compared to the water extract. The results indicated high biological potential in the investigated extracts. Among all extracts, the aqueous extract showed slightly higher antimicrobial potential, especially against Gram-positive strains, probably due to the release of components soluble in water from the dry unexhausted plant material. On the other hand, the acetone and ethanol extracts had significantly higher antioxidative (by 20%), anti-inflammatory activity (up to 3 and 4 times higher, respectively), and α-glucosidase inhibitory potential (3 times higher) than the aqueous extract. The results of this investigation reveal the great potential of the use of *T. montanum* in various branches of food, cosmetics, and the pharmaceutical industry. An important part of this research is a confirmation that, once exhausted by water extraction, for example by hydrodistillation, *T. montanum* plant material can be reused for obtaining valuable products with a wide range of biological activities.

## 1. Introduction

The genus *Teucrium* (Lamiaceae) includes more than 300 species, of which 49 grow in Europe, and they are divided into six sections: *Teucrium*, *Stachybotrys*, *Scorodonia*, *Scordium*, *Chamaedrys*, and *Polium*. Species such as *T. scordium* L., *T. chamaedrys* L., *T. montanum* L., *T. polium* L. spp. *Capitatum*, *T. micropodioides* Rouy., and *T. parviflorum* have been used as traditional medicinal herbs and have been the subject of numerous studies that showed multiple positive health effects from these plants due to their chemical composition [1,2,3]. Literature data have shown that Teucrium species contain different phenolic acids and flavonoids with very strong biological activity, such as antimicrobial, antioxidant, and antihyperglycemic activity [4,5,6].

*Teucrium montanum*, known as iva grass, mountain germander, and mediterranean germander, is a perennial, shrub-like plant up to 25 cm high and blooms from the beginning to the end of summer. This plant inhabits serpentine rocks and thermophilic limestone, edges of forests, and dry mountain meadows in Europe, mostly on the Balkan peninsula and Anatolia in Asia [7,8]. *T. montanum* is widespread in the area of Bosnia and Herzegovina, and it has been traditionally used in folk medicine as a diuretic, stomachic, analgesic, antispasmodic, choleretic, antidiabetic, antirheumatic, antiphlogistic, antiseptic, anthelmintic, carminative, and antipyretic [9,10,11]. It is mostly consumed as tea, soaked in brandy, and mixed with honey. Previous research has shown that *T. montanum* contains biologically active components that are responsible for its antioxidant, antimicrobial, anti-inflammatory, antiproliferative, and proapoptotic activity [4,12,13,14,15]. Qualitative analyses of *T. montanum* have shown the presence of various phenolic compounds such as gallic acid, gentisic acid, chlorogenic and neochlorogenic acid, caffeic acid, protocatechuic acid, p-coumaric acid, vanillic acid, ferulic acid, rosmarinic acid, syringic acid, 3,5-dimetoxy-4-hydroxycinnamic acid, rutin, luteolin and its derivatives, quercetin and its derivatives, diosmetin and its derivatives, catechin, epicatechin, narginin, cirsiliol, cirsimaritin, verbascoside, cynaroside, echinacoside, and samioside [1,4,7]. The harvesting of *T. montanum* (iva grass) on Ozren mountain, Gostilj (Bosnia and Herzegovina), is inscribed (thirteenth session of the Intergovernmental Committee in Port Louis, Republic of Mauritius, 26.11.–1.12. 2018) on the UNESCO Representative List of the Intangible Cultural Heritage of Humanity. It is believed that iva grass has a special medicinal effect when harvested on 11 September, the day of the beheading of St. John the Baptist [16].

A large number of different extraction techniques and different solvents are used for the isolation and separation of bioactive compounds from Teucrium species. In addition to conventional extractions such as maceration, percolation, digestion, and the preparation of decoctions and infusions, other techniques such as microwave-assisted, subcritical water, or ultrasonic extraction have also been used lately. In this research, discontinuous water extraction and combined ultrasonic and discontinuous extraction with ethanol and acetone were performed [5,7,15,17,18]. According to our knowledge, there is no research on *T. montanum* from Ozren mountain, Gostilj, where the picking of this plant is under UNESCO protection. The aim of this study was to determine the content of biologically active components of water, ethanol, and acetone extracts of *T. montanum*. In this sense, the antioxidant, antimicrobial, anti-inflammatory, and antihyperglycemic activities of the obtained extracts were also determined.

## 2. Results and Discussion

### 2.1. Total Polyphenol and Flavonoid Content of T. montanum Extracts

The results of total polyphenols and total flavonoid content in different extracts of iva grass are presented in Table 1.

The acetone extract showed the highest total polyphenol content (TPC), statistically higher (*p* ≤ 0.05) compared to TPC in the aqueous extract. The acetone extract also showed statistically higher total flavonoid content (TFC) (*p* ≤ 0.05) compared to the aqueous and ethanol extracts. Acetone has the lowest dipole moment value among the solvents used, which affects the high content of flavonoids in acetone extract [19]. The aqueous extract had the lowest content of total polyphenols and total flavonoids. According to the literature data, acetone extract had the highest flavonoid content compared to the water, methanol, ethyl acetate, and petroleum ether extracts of *T. montanum* from the Goč mountain in Serbia [13]. The antioxidant capacity of the extracts depends on the solvent used for the extraction process. Polar solvents, such as water and acetone are widely used in extraction of polyphenols which are responsible for antioxidant activity of the plant extracts [20]

The results of TPC and TFC in the aqueous extract were in line with the literature data obtained by Stanković et al. [13] for *T. montanum* from central Serbia and by Bektasevic et al. [8] for *T. montanum* from west Bosnia and Herzegovina. Lower TPC content was found in the aqueous extract of *T. montanum* from West Serbia [4]. In the ethanol extract, TPC was higher and TFC was lower compared to the results obtained by Vujanović et al. [17]. Also, TPC in the acetone extract was higher and TFC was lower compared to the results obtained by Stanković et al. [13,19].

### 2.2. Composition of T. montanum Extracts Obtained by UHPLC-DAD-MS/MS Analysis

**Figure 1:**
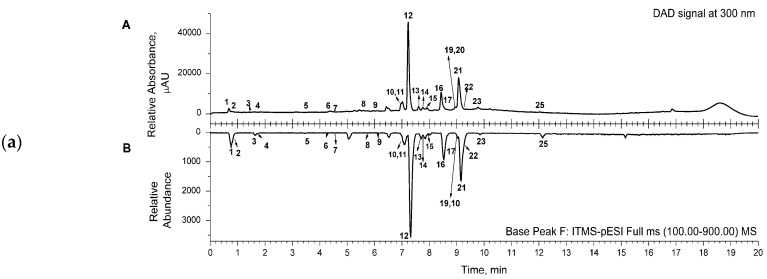

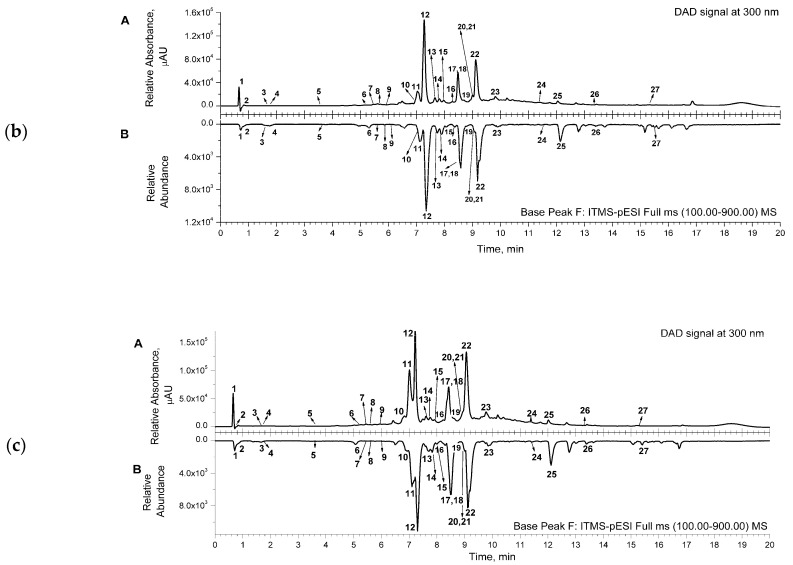
(**a**) Representative UHPLC chromatogram of aqueous extract, recorded from DAD signal at 300 nm (A); mirrored UHPLC chromatogram in range of base peak from MS detection (B). (**b**) Representative UHPLC chromatogram of acetone extract, recorded from DAD signal at 300 nm (A); mirrored UHPLC chromatogram in range of base peak from MS detection (B). (**c**) Representative UHPLC chromatogram of ethanol extract, recorded from DAD signal at 300 nm (A); mirrored UHPLC chromatogram in range of base peak from MS detection (B). Numbers marked under the peaks are in accordance with the same ones given in Table 2 for the detected compounds.

Representative chromatograms (from DAD and MS detectors) are shown in Figure 1. for the aqueous (a) and acetone (b) extracts. The corresponding chromatograms for the ethanol extract were similar to the ones for the acetone extract. Several classes of compounds were detected and identified in the samples of the extracts (Table 2). Three main classes, phenylethanoid glycosides (comp. no. 5, 8, 10–16, 22), phenolic acids and their derivatives (comp. no. 1, 6, 7, 9), and flavonoids (comp. no. 17–21, 23–25), were detected in the extracts. Two octadecadienoic acids (trihydroxy- and hydroxy-octadecenoic acid—comp. no. 26 and 27, respectively) and two flavonoid compounds, luteolin-7-O-glucoside and luteolin (comp. no. 18 and 24, respectively), were detected only in acetone and ethanol extracts (Table 2).

The main constituents of the extracts were phenylethanoid glycosides, such as compounds assigned as caerulescenoside and echinacoside (comp. no. 11 and 12, respectively) and verbascoside and isoverbascoside (comp. no. 16 and 22, respectively), clearly visible in the presented chromatograms (Figure 1).

Similarly, phenylethanoid glycosides were found to be the main constituents of the extracts of *T. montanum* and many *Teucrium* species from North Macedonia extracted by methanol [1]. The composition of the analyzed acetone extract was similar to the results obtained for methanol extracts reported in previous studies [1,7], which can be explained by the same polarity index of acetone and methanol [27]. Verbascoside and echinacoside were the main compounds in the heat-assisted aqueous extracts of *T. montanum* presented by Mandura Jarić et al. [2] and various aqueous extracts of *T. montanum* from Croatia [28]. Verbascoside is one of the most abundant compounds in the ethanol extracts of different *Teucrium* species from Turkey [3], as are caerulescenoside, echinacoside, and verbascoside in the methanol extracts of *T. montanum* from Serbia [7]. Phenylethanoid glycosides are a well-known class of compounds with strong biological and pharmacological activities such as antioxidant, antibacterial, and neuroprotective, as well as anti-aging effects [29,30]. Caerulescenoside is active as an antioxidant by inhibiting LDL oxidation [31]. Echinoside is known as a biomolecule with neuroprotective, anti-inflammatory, and anti-aging effects [30]. Verbascoside is also active as a neuroprotective, antioxidative, antibacterial, anti-inflammatory, and antitumoral compound [32].

In the extracts of commercially available dry plant material of *T. montanum* from Serbia obtained by subcritical water extraction, naringin and gallic acid were the principal detected compounds among phenolic acids such as protocatechuic acid, catechin, chlorogenic acid, vanillic acid, epicatechin, and ferulic acid, while in the extracts presented in this work, quinic acid, p-coumaric acid hexoside, and protocatechuic acid glycoside were detected (Table 2).

Flavonoid compounds such as luteolin and its glycosides, kaempferol glycosides, quercetin glycosides, and diosmetin are common compounds detected in various extracts of *T. montanum* of various origins [1,7,8], as well in the presented paper (Table 2).

**Figure 2:**
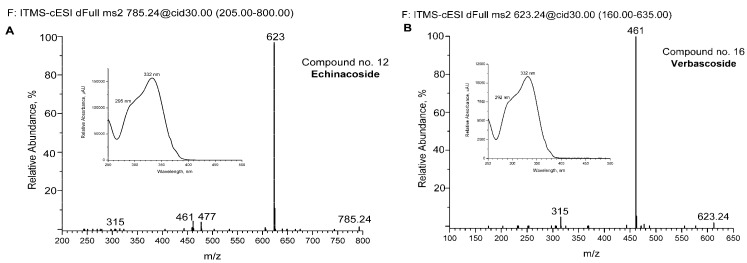

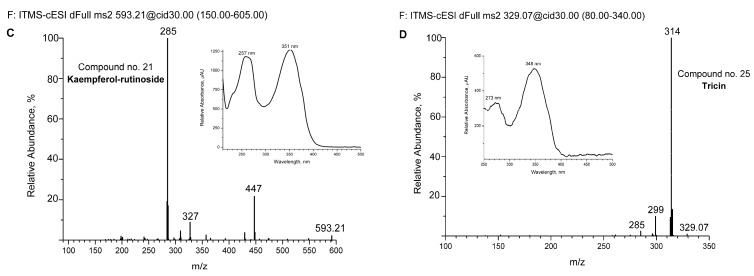
Spectra of some detected compounds eluted from aqueous extract by UHPLC-DAD and UHPLC-MS chromatography. MS/MS spectra of echinacoside (**A**), verbascoside (**B**), kaempferol-rutinoside (**C**), and tricin (**D**). The corresponding UV-Vis absorption spectra are shown in increments. The compounds are numbered as in Figure 1 and Table 2.

For some of the major detected compounds, the corresponding UV-Vis and MS/MS spectra are shown in Figure 2A for two phenylethanoid glycosides (echinacoside and verbascoside—comp. no. 12 and 16, respectively) and two flavonoids (kaempferol-rutinoside and tricin—comp. no. 21 and 25, respectively).

### 2.3. The Antimicrobial Activity of the T. montanum Extracts

Plant-derived bioactive compounds are widely known for their high antioxidant and antimicrobial potential [33,34]. Analysis of antimicrobial activity indicated similar antimicrobial potential for all the analyzed extracts (Table 3). All extracts exhibited substantial antimicrobial activity against all analyzed microorganisms. The antibacterial activity can be considered higher than antifungal, probably due to the composition of the extracts. The observed antimicrobial activity probably relies on the presence of phenylethanoid glycosides such as caerulescenoside, echinacoside, verbascoside, and isoverbascoside, which are proven to be potent antimicrobial agents [29,30,32]. The aqueous extract had a higher antimicrobial effect against Gram-positive strains compared to Gram-negative strains. Additionally, this extract showed slightly higher antimicrobial potential compared to the ethanolic and acetone extracts. This can be explained by the contact of water with unexhausted plant material, which was not the case for the ethanol and acetone extracts. The aqueous extract had fewer compounds compared to the other two extracts, but that did not reflect its antimicrobial activity due to the fact that the synergistic effect of different bioactive compounds has much stronger antimicrobial potential compared to pure bioactive compounds [33]. Besides the presence of some specific compounds, their concentration can significantly affect the biological activity. This can explain slight differences in the antimicrobial activity of the ethanol and acetone extracts, considering the fact that the qualitative composition of the extracts was the same. Different levels of antimicrobial activity in *T. montanum* extracts have been reported for different solvents, regardless of the similarity of their composition [7]. Additionally, the type of solvent used for the extraction influences the distribution and amount of phenolic compounds and, consequently, the antimicrobial activity of the extracts [35]. The obtained results of the research are quite opposite to the results of the investigation of different extracts of *T. montanum* reported by Djilas et al. [18], in which water extract had no antimicrobial activity against seven tested microorganisms, including *E. coli*, *P. aeruginosa*, and *St. aureus.* Also, extracts of *T. montanum* showed a broader spectrum of antimicrobial activity compared to extracts of *T. polium*’s aerial part and root [28]. A previous examination of acetone extract of *T. montanum* from Serbia and Montenegro showed much lower antimicrobial activity against the same ATCC strains of *E. coli* and *St. aureus* compared to the presented results [14].

### 2.4. Antioxidative Activity, Anti-Inflammatory Activity, and α-Glucosidase Inhibitory Potential of T. montanum Extracts

The results of antioxidative activity, anti-inflammatory activity, and α-glucosidase inhibitory potential of *T. montanum* extracts are presented in Table 4. Among the analyzed extracts, the acetone extract showed the highest capacity to neutralize DPPH radicals, while the aqueous extract showed the lowest. This can be explained by the highest content of total polyphenols and total flavonoids being detected in the acetone extract and the lowest content of these components being present in the aqueous extract (Table 1). The differences between the antioxidant activities of the analyzed extracts were statistically significant (*p* ≤ 0.05). The results of antioxidant activity were slightly lower compared to the results of the aqueous extract, but higher compared to the results of the acetone extract, of *T. montanum* obtained by Stanković et al. [13]. Bektasevic et al. [8] obtained similar, and Djilas et al. [18] obtained lower, antioxidant activity for *T. montanum* aqueous extract compared to the results presented in this study. The antioxidant activity established for the ethanol extract in this study was higher than the results presented by Oalđe et al. [5].

Tissue protein denaturation occurs during the inflammatory process, which is a common risk factor for the development of diseases such as infections, arthritis, type 2 diabetes mellitus, obesity, cancer, etc. [36]. The ethanol extract had significantly higher (*p* ≤ 0.05) anti-inflammatory activity compared to the acetone and aqueous extracts. The results of the anti-inflammatory activity of the extracts (25 mg/mL) were higher for ethanol, equivalent for acetone, and lower for aqueous extract compared to the positive control diclofenac sodium (0.25 mg/mL). The aqueous extracts, with the lowest TPC and TFC, also had the lowest anti-inflammatory activity. Dharmadeva et al. [37] suggested that polyphenolic compounds, flavonoids, and tannins, individually or in combination, have an anti-inflammatory effect. Consistent with our results, anti-inflammatory activity has been found for different plants, such as *Satureja kitaibelii* Wierzb. ex Heuff. [36], *Ficus racemosa* L. [37], and *Mikania scandens* L. wild [38]. Bektasevic et al. [8] established the protective effect of *T. montanum* on proteins.

Enzyme α-glycosidase is responsible for the absorption of digested glucose from starch and other polysaccharides in the small intestine, resulting in increased blood glucose levels. The inhibition of this enzyme can help to reduce postprandial glucose load in the blood by slowing down the breakdown of polysaccharides into glucose. The ethanol and acetone extracts showed significantly higher (*p* ≤ 0.05) α-glucosidase inhibitory potential compared to the aqueous extract (Table 4). The α-glucosidase inhibitory activity of the ethanolic and acetone extracts (25 mg/mL) was equivalent to the positive control acarbose (0.0005 mg/mL), and the inhibitory activity of the aqueous extract was significantly lower. The aqueous extract had the lowest content of total polyphenols and total flavonoids compared to the other two extracts (Table 1). Polyphenol compounds, such as flavonoids, exhibit significant antidiabetic effects by inhibiting the activity of certain enzymes [39,40]. Proenca et al. [41] found that quercetin and luteolin have a strong ability to inhibit a-glucosidase due to the presence of the OH group in the C-ring in position 3. The aqueous extract of *T. montanum* did not contain luteolin (Table 2). The ethanol extract with lower TPC and TFC showed slightly higher (*p* ≥ 0.05) α-GIP inhibitory potential compared to the acetone extract. Kidane et al. [42] found that in addition to polyphenols, some other components, such as saponins and alkaloids, may have inhibitory effects against α-glucosidase. Pavlović et al. [43] found that ethanolic and aqueous extracts of *T. montanum* at a concentration of 0.50 mg/mL inhibited 11.24% and 11.97% of α-glucosidase enzyme, which is more comparable to the results obtained in this study. Vujanović et al. [17] found that aqueous extracts of different Teucrium species inhibited the enzymes α-glucosidase and α-amylase, whereby the extract of T. montanum showed higher inhibition values compared to *T. chamaedrys*.

## 3. Materials and Methods

### 3.1. Plant Material

*T. montanum* was harvested on 11 September 2020 on Ozren mountain, Gostilj, Bosnia and Herzegovina (altitude: 700 m; latitude: 44.66074; longitude: 18.19920). Identification of the plant material was performed by professor Svjetlana Zeljković, PhD (Faculty of Agronomy, University of Banja Luka, B&H).

The sample was dried outdoors in the air without direct exposure to sunlight and then stored in the dark at room temperature until extract preparation.

### 3.2. Extract Preparation

Sample extractions were performed according to the method described by Oalđe et al. [5] with some modifications. The aerial part of the plant was used to prepare the extracts. Dried aerial parts of *T. montanum* (250 g) were extracted for 3 h with 500 mL of boiling water (100 °C). The obtained extracts were evaporated to dryness in a rotary vacuum evaporator at temperatures up to 60 °C. After isolating the aqueous extract, *T. montanum* was dried and used to obtain ethanol and acetone extracts. A mixture of *T. montanum* (50 g) and 250 mL of 80% ethanol was placed in ultrasonic bath for 5 min. After that, a discontinuous extraction was performed at boiling temperature for 1 h. Subsequently, ethanol extract was filtered and dried. The same procedure was repeated with 60% acetone to obtain an acetone extract. The extracts were stored in glass vials at −20 °C until analysis.

### 3.3. Determination of Total Polyphenol and Flavonoid Content

The total polyphenol content (TPC) of T. montanum extracts was determined in accordance with Odžaković et al. [44] by the Folin–Ciocalteu method. The content of polyphenolic compounds was determined by measuring the absorbance with a UV–vis spectrophotometer (Lambda 25, PerkinElmer, Shelton, CT, USA), and the results were expressed as mg of gallic acid equivalent per gram of dry extract (mg GAE/g DW).

A method reported by Orsavová et al. [45] was used for the evaluation of total flavonoid content (TFC), and the results were expressed as mg of quercetin equivalent per gram of dry extract (mg QE/g DW).

### 3.4. UHPLC Analysis of the T. montanum Extracts

Liquid chromatography with ultra-high performance (UHPLC) analysis, in accordance with Zvezdanović [46], was used for a qualitative analysis of the extracts. A Dionex Ultimate 3000 UHPLC+ system equipped with a diode array (DAD) detector and LCQ Fleet Ion Trap Mass Spectrometer (Thermo Fisher Scientific, Waltham, MA, USA) with a Hypersil gold C18 column (50 × 2.1 mm, 1.9 μm) was used for determination. The column was tempered at 25 °C with a mobile phase composed of two solvents, 0.1% formic acid in water (A) and methanol (B) at a 0.250 mL/min flow rate. Three detection wavelengths set at 300, 330, and 350 were used for DAD signal estimation. Mass spectrometric analysis was performed using a 3D ion trap with electrospray ionization (ESI) in negative ionization mode. MS spectra were acquired by full-range acquisition at *m*/*z* 100–900, with a tandem mass spectrometry analysis performed by a data-dependent scan—the collision-induced dissociation of detected molecular ions peaks ([M–H]−) tuned at 30 eV. For instrument control, data acquisition, and data analysis, Xcalibur software (version 2.1) was used. Data were further processed with Origin 7.5 software. The assignation of the detected compounds was based on their retention times, UV–Vis spectra from the DAD detector, and MS spectra with the corresponding molecular ion peaks, as well as the characteristic ion fragmentation of selected peaks (MS/MS) from the corresponding UHPLC chromatograms. Identification of the detected compounds was performed according to reference standards for some compounds. The corresponding data were compared with the data available in the literature.

### 3.5. Determination of Antimicrobial Activity of T. montanum Extracts

The antimicrobial activity was expressed as the values of minimal inhibitory concentration (MIC) and minimal bactericidal concentration (MBC). The determination was performed according to the method described by Balouiri et al. [47] against four Gram-positive strains (*Bacillus subtilis* ATCC 6633, *Bacillus cereus* ATCC 11778, *Listeria monocytogenes* ATCC 15313, and *Staphylococcus aureus* ATCC 25923) and four Gram-negative strains (*Escherichia coli* ATCC 25922, *Proteus vulgaris* ATCC 8427, *Pseudomonas aeruginosa* ATCC 27853, and *Klebsiella pneumoniae* ATCC 700603). Antimycotic activity was determined against one yeast, *Candida albicans* ATCC 2091, and two mold strains, *Aspergillus niger* and *Penicillium* sp. The extracts were diluted in the range from 0.04 to 20 mg/mL and added to microtiter plates inoculated with the appropriate microbial strain. The microtiter plates were incubated at 37 °C for 24 h for bacteria and 72 h at 25 °C for fungi. The results were determined by an EZ read 400 Elisa microplate reader (Biochrom, UK). The extracts at concentrations which did not show any growth of the tested microorganisms were plated to Mueller–Hinton agar (“Torlak”, Belgrade, Serbia) for bacteria and Sabouraud maltose agar (“Torlak”, Belgrade, Serbia) for fungi, and incubated for 48 h at 37 °C and 5 days at 25 °C for bacterial and fungal strains, respectively. The lowest concentration which indicated no growth was recorded as the MBC value.

### 3.6. Determination of Antioxidant Activity of T. montanum Extracts

An assessment of the antioxidant activity of the extracts was performed by evaluating the free radical scavenging effect on the 2,2-diphenyl-1-picrylhydrazyl (DPPH) radical, according to Odžaković et al. [44]. The antioxidant activity of the samples was expressed as IC50 (mg/mL).

### 3.7. Determination of Anti-Inflammatory and α-Glucosidase Inhibitory Potential of T. montanum Extracts

A protein denaturation bioassay described by Ullah et al. [48] was used for the determination of anti-inflammatory activity (AIA). A total volume of 2 mL of extract with a concentration of 25 mg/mL was incubated with 0.2 mL of egg albumin and phosphate-buffered saline (pH 6.4) at a temperature of 37 °C for 15 min and at 70 °C for 5 min. The mixture was then cooled down and the absorbance at 660 nm was measured. AIA was expressed as percent of the protein denaturation inhibition. Diclofenac sodium (0.25 mg/m) was used as a positive control with an AIA 47.67%.

α-glucosidase inhibitory potential (α-GIP) was determined by a method described by Tumbas Šaponjac et al. [49], where each well contained 100 µL of 2 mmol/L 4-nitrophenyl α-D-glucopyranoside in 10 mmol/L potassium phosphate buffer (pH 7.0) and 20 µL of the samples (25 mg/mL) diluted in buffer. An amount of 100 µL of the enzyme solution (56.66 mU/mL) was used for the initiation of the reaction. The plates were then incubated at 37 °C for 10 min and the absorbance at 405 nm was measured. According to the results, α-GIP (%) values were calculated and were compared with the positive control acarbose (0.0005 mg/mL) with an α-GIP of 35.42%.

### 3.8. Statistical Analysis

Statistical analysis was performed by the software Statistica 12.0 (StatSoft, Inc., Tulsa, OK, USA) using one-factor analysis of variance (ANOVA). The values of the results were presented as mean values with standard deviations (SDs). The significance level of differences between mean values at *p* < 0.05 was determined by Duncan’s test.

## 4. Conclusions

In general, the obtained results of the investigation of aqueous, acetone, and ethanol extracts of *T. montanum* emphasize their great potential for use in the food, pharmaceutical, and cosmetic industries. All extracts represent a rich source of polyphenolic compounds, which are responsible for antioxidative, anti-inflammatory, and α-glucosidase inhibitory potential. Additionally, substantial antimicrobial activity against bacterial and fungal strains was noticed for all the analyzed extracts. Nevertheless, the fact that acetone and ethanol extracts exhibited a range of biological activities opens the possibility of investigating the reuse of exhausted plant material that remains after the preparation of aqueous extracts, or the isolation of essential oil not only from *T. montanum* but also from various plant species.

## Figures and Tables

**Table 1:** Content of total polyphenols (TPC) and total flavonoids (TFC) in *T. montanum* extracts.

Samples	TPC (mg GAE/g DW)	TFC (mg QE/g DW)
Aqueous extract	109.11 ^b^ ± 4.41	14.37 ^c^ ± 0.45
Acetone extract	134.81 ^a^ ± 2.31	20.76 ^a^ ± 0.51
Ethanol extract	129.20 ^a^ ± 4.72	18.78 ^b^ ± 0.44

The results are expressed as mean value (n = 3) ± standard deviation. Mean values with different superscript letters in the same column have a statistically significant difference with 95% probability (*p* < 0.05).

**Table 2:** Qualitative composition of aqueous (Aq), acetone (Ac), and ethanol (Et) extracts of *T. montanum* obtained by UHPLC-DAD-MS/MS analysis.

Peak No.	tR, Min	UV/Vis Data from UHPLC-DAD Signal Absorb. Max., nm	Molecular Ion [M–H]−*m*/*z*	MS/MS Fragment Ions	Assignment (Reference)	Sample		
						Aq	Ac	Et
1	0.79	-	191	173,127,111, 85 (100%)	quinic acid [21]	+	+	+
2	0.92	-	191	173,111 (100%)	citric acid (standard)	+	+	+
3	1.64	-	191	129,101 (100%)	(n.i.)	+	+	+
4	1.80	-	191	129,101 (100%)	(n.i.)	+	+	+
5	3.42	331 300 sh	623	477/478 (100%), 461	forsythoside A [22]	+	+	+
6	5.30	-	535	489,327,309,179,163 (100%)	n.i. phenolic acid derivative	+	+	+
7	5.50	-	325	265,163 (100%), 119	p-coumaric acid hexoside [23]	+	+	+
8	5.83	-	607	461 (100%), 315	lipedoside A [22,24]	+	+	+
9	6.10	-	299	153 (100%)/152,109	n.i. protocatechuic acid-glycoside	+	+	+
10	7.09	331 294 sh	639	621 (100%), 529,459	β-hydroxyverbascoside diastereoisomer [25]	+	+	+
11	7.10	332 290 sh	785	623 (100%), 477,461,315	caerulescenoside [1]	+	+	+
12	7.33	332 295 sh	785	623 (100%), 477,461,315	echinacoside [1]	+	+	+
13	7.74	330 289 sh	639	621 (100%), 529,459	β-hydroxyverbascoside diastereoisomer [25]	+	+	+
14	7.91	327 300 sh	639	621 (100%), 529,459	β-hydroxyverbascoside diastereoisomer [25]	+	+	+
15	8.01	332 296 sh	799	623 (100%), 605,477,305	castanoside [1]	+	+	+
16	8.51	332 292 sh	623	461 (100%), 315	verbascoside [22,25]	+	+	+
17	8.68	266 357	593	447,285 (100%)	luteolin-rutinoside [22]	+	+	+
18	8.70	262 354	477	-	luteolin-7-O-glucoside (standard)	-	+	+
19	8.96	-	463	300/301 (100%), 271,255,179,151	isoquercitrin (quercetin-3-O-glucoside) (standard)	+	+	+
20	8.98	-	609	591,463,343, 300/301 (100%), 271	rutin (quercetin-3-O-rutinoside) (standard)	+	+	+
21	9.03	257 351	593	447,327,285 (100%)	kaempferol-rutinoside [26]	+	+	+
22	9.25	331 290 sh	623	461 (100%), 315	isoverbascoside [22,25]	+	+	+
23	9.91	251 340	607	299 (100%), 284	diosmin (* MB: FIO01056)	+	+	+
24	11.46	-	285	267,243,217 (100%)	luteolin (standard)	-	+	+
25	12.14	273 348	329	314 (100%)/315/313,299/300,285	tricin (* MB: FIO00745)	+	+	+
26	13.39	-	329	314,293,229 (100%), 211,171	trihydroxy-octadecenoic acid [15]	-	+	+
27	15.40	-	295	277 (100%), 195,183,171	hydroxy-octadecadienoic acid [15]	-	+	+

*—https://massbank.eu/MassBank/Search, accessed on 16 January 2024; n.i.—not identified; sh—shoulder.

**Table 3:** Antimicrobial activity of *T. montanum* extracts expressed as minimal inhibitory concentration (MIC) and minimal bactericidal concentration (MBC), expressed in mg/mL.

Antimicrobial Activity, mg/mL	Aqueous Extract		Ethanol Extract		Acetone Extract	
	MIC	MBC	MIC	MBC	MIC	MBC
Gram-positive bacteria						
*Staphylococcus aureus* ATCC 25923	1.25	10	5	20	5	20
*Bacillus cereus* ATCC 11778	5	20	2.5	20	2.5	20
*Listeria monocytogenes* ATCC 15313	1.25	5	2.5	20	1.25	>20
*Bacillus subtilis* ATCC 6633	5	>20	5	20	5	20
Gram-negative bacteria						
*Escherichia coli* ATCC 25922	5	20	5	10	5	20
*Proteus vulgaris* ATCC 8427	2.5	5	2.5	20	2.5	>20
*Pseudomonas aeruginosa* ATCC 27853	2.5	20	2.5	5	2.5	>20
*Klebsiella pneumoniae* ATCC 700603	1.25	20	1.25	10	1.25	20
Fungus						
*Candida albicans* ATCC 2091	5	10	5	20	5	20
*Aspergillus niger*	2.5	20	2.5	20	2.5	20
*Penicillium* sp.	1.25	20	2.5	20	2.5	20

**Table 4:** Antioxidative activity (DPPH), anti-inflammatory activity (AIA), and α-glucosidase inhibitory potential (α-GIP) of *T. montanum* extracts.

Samples	DPPH (IC_50_ (µg/mL))	AIA (%)	α-GIP (%)
Aqueous extract	35.70 ^a^ ± 0.02	13.12 ^c^ ± 2.12	10.97 ^b^ ± 1.04
Acetone extract	28.13 ^c^ ± 0.01	44.82 ^b^ ± 3.23	31.52 ^a^ ± 2.37
Ethanol extract	28.22 ^b^ ± 0.05	58.01 ^a^ ± 3.66	32.54 ^a^ ± 2.50

The results are expressed as mean value (n = 3) ± standard deviation. Mean values with different superscript letters in the same column have a statistically significant difference with 95% probability (*p* < 0.05).

## Data Availability

Data are contained within the article.
